# Performance Enhancement of Piezoelectric Single Crystals Through Combination of Alternating-Current Poling and Direct-Current Poling

**DOI:** 10.3390/s26010140

**Published:** 2025-12-25

**Authors:** Chenyang Zheng, Hao Wang, Jinpeng Ma, Bingzhong Shen, Rui Zhang, Xudong Qi, Yang Liu

**Affiliations:** 1 Functional Materials and Acousto-Optic Instruments Institute, School of Instrumentation Science and Engineering, Harbin Institute of Technology, Harbin 150080, China; 21b901031@stu.hit.edu.cn (C.Z.); 22b901035@stu.hit.edu.cn (H.W.); 20240251@hit.edu.cn (J.M.); 20b901042@stu.hit.edu.cn (B.S.); 2 Key Laboratory for Photonic and Electronic Bandgap Materials, Ministry of Education, School of Physics and Electronic Engineering, Harbin Normal University, Harbin 150025, China; xudong-qi@hrbnu.edu.cn; 3 Tianjin Research Institute for Water Transport Engineering, Ministry of Transport, Tianjin 300456, China; liuyang@tiwte.ac.cn

**Keywords:** alternating-current poling, direct-current poling, single crystals, PMN-PT, domain structure, ultrasound transducer

## Abstract

**Highlights:**

**What are the main findings?**

- A new approach to polarize the PMN-PT single crystals was proposed in the work.
- There were two optimal polarization windows in the new approach experiment’s result.

**What are the implications of the main findings?**

- The multi-peak phenomenon needs to be explained in theory.
- The performance enhancement of PMN-PT single crystals can be customized in this way.

**Abstract:**

Alternating-current poling (ACP) is becoming a mainstream method because of its stronger ability in promoting the piezoelectric performance of ferroelectric single crystals than that of direct-current poling (DCP). A novel approach was developed by incorporating alternating-current poling and direct-current poling as modified alternating-current poling (MACP). According to the comparison of performance differences between AC-poled and DC-poled single crystals, the properties of MACP single crystals under specific conditions were systematically investigated. The improvement of single crystal performance by MACP is manifested by the multi-peak increase in piezoelectric coefficient ($d_{33}$) and relative dielectric permittivity ($\varepsilon_{33}^{T}/\varepsilon_{0}$), and the coupling factor ($k_{t}$) value under higher DC bias is higher than that under DC polarization, rather than a direct superposition of DCP and ACP. Two optimal polarization windows were found: 0.2–0.25 kV/mm and 0.35–0.6 kV/mm. Compared with DCP, MACP increases the $d_{33}$, $\varepsilon_{33}^{T}/\varepsilon_{0}$ and $k_{t}$, of single crystals by up to 45.67%, 21.62%, and 24.54%, respectively. This significant performance improvement, combined with its complexity, provides a new direction for customizing the performance of single crystals.

## 1. Introduction

Piezoelectric materials, which enable the direct conversion of energy between mechanical and electrical forms, are integral to a wide range of technologies, including ultraprecise actuators, sensors, transducers, and nonvolatile memory devices. In recent years, significant research attention has been paid toward relaxor-based ferroelectric single crystals, like Pb(Mg_1/3_Nb_2/3_)O_3_-PbTiO_3_ (PMN-PT), for their exceptional piezoelectric performance [1]. Over the past decades, strategies, such as doping modification [2,3,4], phase structure design [5], and domain engineering [6,7,8], have been employed to enhance the electromechanical properties of piezoelectric materials. Among these, domain engineering has proven particularly effective in unlocking the full potential of piezoelectric materials by deliberately designing their internal domain configurations.

Recent studies highlight the efficacy of the alternating current poling (ACP) technique in this regard. For instance, Yamamoto et al. demonstrated that ACP enhances electromechanical performance by increasing domain wall density and introducing controlled heterogeneity into the domain structure [9,10]. Wada et al. achieved superior piezoelectricity in BaTiO_3_ single crystals by refining domain size through tailored poling protocols [11]. In PMN-0.25PT single crystals, Xu et al. reported a 40% increase in the piezoelectric coefficient ($d_{33}$) using ACP [12,13]. Furthermore, Wan et al. investigated the influence of temperature on ACP outcomes, finding that PMN-0.3PT single crystals poled at 343 K exhibited optimal values for $d_{33}$, $d_{31}$, and $\varepsilon_{33}^{T}/\varepsilon_{0}$, correlated with a maximized domain wall density under these specific conditions [14]. Qiu et al. also observed 20–30% improvements in $d_{33}$ and $\varepsilon_{33}^{T}/\varepsilon_{0}$ in relaxor-PbTiO_3_ single crystals via ACP and established a relationship between sample thickness and the resulting electromechanical and dielectric properties [15]. Li et al. demonstrated that La-modified BLMT relaxor-ferroelectric films exhibited significantly enhanced fatigue resistance—surviving over 10^8^ cycles with <3% degradation—via ACP, and established the relationship between nanodomain switching pathways (bipolar vs. multi-step) and macroscopic polarization behavior through in situ PFM and thermodynamic modeling [7].

Previous investigations indicated that the piezoelectric performance of relaxor-ferroelectric single crystals is significantly influenced by electric poling conditions. This is exemplified by the construction of electric-field–temperature–composition phase diagrams in typical relaxor-PT systems, which have provided fundamental insights into the effects of direct-current (DC) poling [16]. The strategic engineering of domain structures through the fine-tuning of key poling parameters [17] has been established as an effective pathway to achieving a superior piezoelectric response.

Given that ACP can significantly improve the performance of single crystals and DCP can also maintain the performance of single crystals at a high level, according to the studies mentioned above, the present study not only systematically studied the effects of changing key process parameters of ACP, such as voltage amplitude, frequency, and polarization cycle times, on the performance of PMN-PT single crystals, but also combined traditional ACP with DCP and used AC polarization with an added DC bias as an improvement to AC polarization, to study the effect of MACP on the performance of PMN-PT single crystals.

## 2. Materials and Experiment

The PMN-PT single crystal samples (supplied by the Shanghai Institute of Ceramics, Chinese Academy of Sciences) were sectioned into specimens with dimensions of 2 × 2 × 1 mm^3^. The samples had a nominal composition of PMN-0.3PT. Gold electrodes were subsequently sputtered onto the major surfaces of the specimens.

For DCP, samples were poled under a DC field of 1.5 kV/mm for 30 min in silicone oil at room temperature. For the ACP treatment, a symmetrical triangular waveform of a certain Vpp, frequency, and number of cycles or bias was applied using a Function/Arbitrary Waveform Generator (Agilent Technologies, Santa Rosa, CA, USA), and the signal was amplified through a high-voltage amplifier (Trek Model 10/40A, Trek, Inc., Lockport, NY, USA).

The relative dielectric constant $\varepsilon_{33}^{T}/\varepsilon_{0}$ was determined from the parallel capacitance approximation measured at 1 kHz using an Agilent 4294A impedance analyzer (Agilent Technologies, Santa Rosa, CA, USA). The thickness electromechanical coupling coefficient $k_{t}$ was derived from impedance resonance spectra following the IEEE standard [18]. The piezoelectric coefficient $d_{33}$ was characterized using a quasi-static ZJ-2 $d_{33}$ meter (Institute of Acoustics, Chinese Academy of Sciences, Beijing, China). All electrical properties were measured after a 24 h aging period following poling.

At the final stage of the experiment, the ferroelectric hysteresis (P-E) loops were measured under a 1 Hz symmetric triangular waveform to evaluate the ferroelectric states achieved by the different poling processes. A direct comparison between the loops of the AC- and MAC-poled samples was conducted to demonstrate the superiority of the modified MACP protocol. It should be noted that due to instrumental limitations, the maximum amplitude of the applied electric field was confined to ±15 kV/cm. Consequently, the obtained hysteresis loops and the derived polarization parameters represent the material’s performance under this sub-saturation electric field condition, rather than its intrinsic saturation properties. The ‘number of cycles’ refers to the complete periods of the symmetrical triangular AC voltage waveform applied at a fixed frequency of 1 Hz.

**Figure 1:**
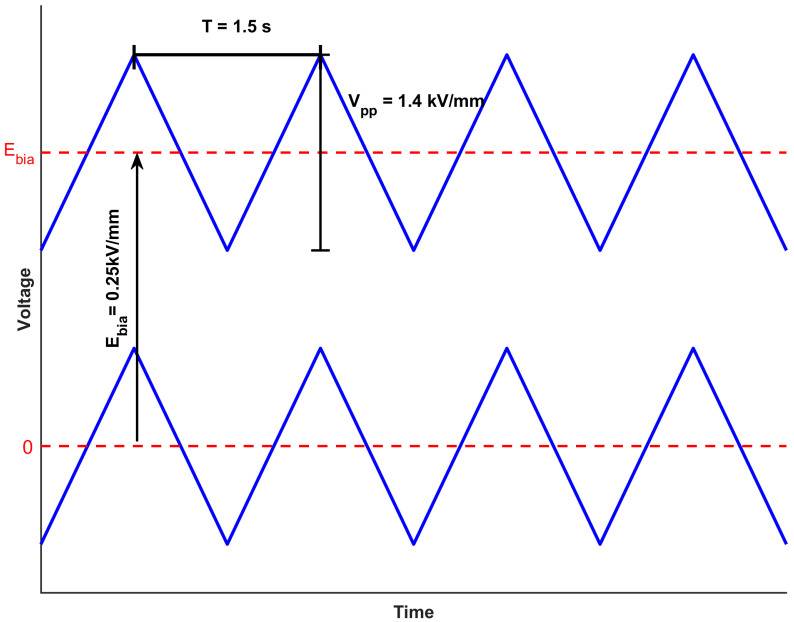
MACP waveform compared with ACP waveform.

Figure 1 shows the waveform used to poling the single crystal in MACP, compared with the waveform of ACP. The ACP triangular wave biased with a certain voltage was used to polarize the samples for MACP experiments, while still keeping the duty cycle at a constant 50%. We anticipated that employing this specific waveform for poling PMN-PT single crystals would not only maintain the performance enhancements achieved through ACP but also lead to further improvements.

To demonstrate the performance differences between AC polarization and DC polarization visually, the piezoelectric constants $d_{33}$ and dielectric constant $\varepsilon_{33}^{T}/\varepsilon_{0}$ and coupling factors $k_{t}$ were normalized by the same factors measured in DCP experiments in this paper.

## 3. Results and Discussion

To validate the ACP behavior of PMN-PT single crystals and compare their performance with that of the DCP, we conducted experiments with the number of cycles, frequency, and AC field amplitude as variables. Finally, we performed MACP experiments with DC bias as the variable. The experimental results are shown in this section. The DCP result used for normalization in this work is $d_{33}$ = 843 pC/N, $k_{t}$ = 0.272934, ${\left(\varepsilon_{33}^{T}\right)}^{DC}/\varepsilon_{0}$ = 1.961133 × 10^3^.

### 3.1. Polarization Cycles

**Figure 2:**
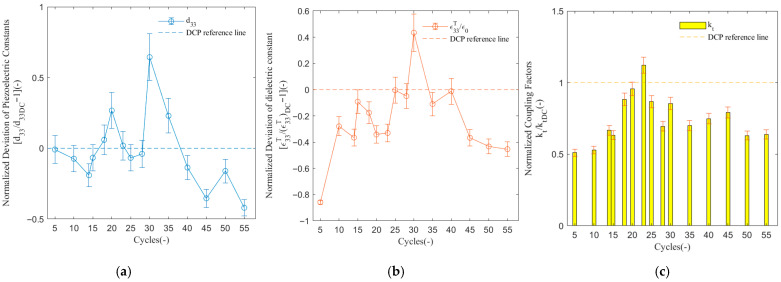
Dependence of piezoelectric and dielectric properties on the number of polarization cycles (N) for the PMN-PT signal crystal at 1 Hz with the Vpp value of 1 kV/mm. (**a**) Evolution of the piezoelectric coefficient $d_{33}$ vs. cycles; (**b**) evolution of the dielectric constant $\varepsilon_{33}^{T}/\varepsilon_{0}$ vs. cycles; (**c**) evolution of the coupling coefficient $k_{t}$ vs. cycles.

Figure 2 shows the normalized deviation of piezoelectric constants $d_{33}$ (ND$d_{33}$, $d_{33}/d_{33}^{DC}-1$) and free dielectric constant $\varepsilon_{33}^{T}/\varepsilon_{0}$ (ND$\varepsilon_{33}^{T}/\varepsilon_{0}$, $\left(\varepsilon_{33}^{T}/\varepsilon_{0}\right)/{\left(\varepsilon_{33}^{T}/\varepsilon_{0}\right)}^{DC}-1={\varepsilon_{33}^{T}/\left(\varepsilon_{33}^{T}\right)}^{DC}-1$), and normalized coupling factors $k_{t}$ (N$k_{t}$, $k_{t}/k_{t}^{DC}$) of the PMN-PT signal crystal under different cycles(N) of the ACP method at the frequency of 1 Hz and at the Vpp electric field of 1 kV/mm.

Three parameters initially increased with the number of cycles. In N = 20 and N = 30, $d_{33}$ surged by 29.77% (from 0.2669 at cyc = 20 to 0.6441 at cyc = 30), indicating large-scale and unstable rearrangement of the domain structure inside the material in the stage [7], accompanied by significant thermal effects and even microdamage [19]. At N = 30, $d_{33}$ and $\varepsilon_{33}^{T}/\varepsilon_{0}$ reached their maximum values, exceeding the DCP results by 64.61% (0.6461) and 43.45% (0.4345), respectively. Meanwhile, $k_{t}$ achieved peak value at N = 23, showing a 12.09% (1.1209) improvement. Therefore, the cycle range between 23 and 30 constitutes the optimal performance window. Beyond this optimal cycle (cyc > 30), all performance parameters exhibited a significant decreasing trend. For instance, $d_{33}$ plummeted by 64.72% from its peak value between N = 30 and N = 55 (from 0.6441 to −0.1601). The discrepancy in the cycle-dependent peaks between $d_{33}$/$\varepsilon_{33}^{T}/\varepsilon_{0}$ and $k_{t}$ is attributed to their different sensitivities to domain wall density and macroscopic domain orientation, respectively. The minor shift in the $k_{t}$ peak falls within the typical measurement uncertainty (±2–3%). This result clearly demonstrates the detrimental effects of over-poling, which is attributed to excessively long duration of ACP breaking the favorably aligned domain in the existing structure [13,20,21], and leading to performance degradation.

Overall, in this dataset, $d_{33}$ and $\varepsilon_{33}^{T}/\varepsilon_{0}$ showed an obvious positive correlation, with their variation trends highly synchronized—both peaking simultaneously at N = 30 and degrading together thereafter. This aligns with the fundamental physics of piezoelectric materials. Both $d_{33}$ and $\varepsilon_{33}^{T}/\varepsilon_{0}$ directly depend on the quantity and quality of domains aligned along the poling direction. When ACP effectively promotes more domain switching, both parameters are enhanced synchronously. $k_{t}$ is partially correlated with both $d_{33}$ and $\varepsilon_{33}^{T}/\varepsilon_{0}$, but exhibited a phase shift. While the overall trend of $k_{t}$ also followed an initial increase, followed by a decrease, similar to $d_{33}$/$\varepsilon_{33}^{T}/\varepsilon_{0}$, its peak (at N = 23) occurred earlier than that of $d_{33}$ and $\varepsilon_{33}^{T}/\varepsilon_{0}$ (at N = 30). This suggests that $k_{t}$ (the electromechanical coupling coefficient) is highly sensitive to specific patterns of the domain configuration. It is inferred that the domain structure reached an optimal configuration for energy conversion efficiency (potentially dominated by 180° domain wall motion) at N = 23. Subsequently, the switching of the more non-180° domains continuously enhanced $d_{33}$ and $\varepsilon_{33}^{T}/\varepsilon_{0}$, but might have introduced defects the more sensitive to energy loss, preventing $k_{t}$ from maintaining its peak value. This research reveals that the poling process occurs in distinct stages, with different stages optimizing corresponding performance aspects.

### 3.2. Polarization Frequency

**Figure 3:**
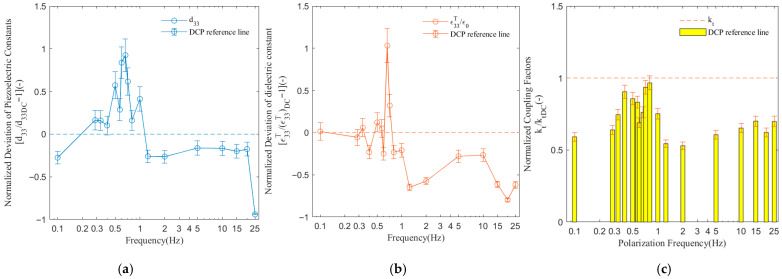
Dependence of piezoelectric and dielectric properties on the AC-poling frequency under fixed conditions of 1 kV/mm and 20 cycles. (**a**) Evolution of the piezoelectric coefficient $d_{33}$ with frequency; (**b**) evolution of the dielectric constant $\varepsilon_{33}^{T}/\varepsilon_{0}$ with frequency; (**c**) evolution of the coupling coefficient $k_{t}$ with frequency.

Figure 3 shows the ND$d_{33}$, ND$\varepsilon_{33}^{T}/\varepsilon_{0}$, and N$k_{t}$ of the PMN-PT signal crystal with different polarization frequencies of the ACP method at 20 cycles and a Vpp electric field of 1 kV/mm.

At a frequency of 0.66667 Hz, $d_{33}$ and $\varepsilon_{33}^{T}/\varepsilon_{0}$ reached their maximum values, exceeding the DCP results by 92.4% (0.9240) and 103.1% (1.0310), respectively. The $k_{t}$ value remained close to the DCP level within the 0.3–0.8 Hz range, peaking at 0.8 Hz. In the 0.5–0.75 Hz interval, its performance was significantly higher than that of DCP. However, for frequencies equal to or greater than 1 Hz, the performance became inferior to DCP and exhibited a steady decline. When the ACP frequency was 1 Hz, the poling effect generally equaled or exceeded that of DCP, with overall higher values but noticeable fluctuations. Performance degraded markedly at frequencies above 1 Hz. When the poling frequency exceeded 0.8 Hz, $k_{t}$ decreased significantly, reaching its lowest point at 2 Hz, followed by a slight recovery with minor fluctuations thereafter. This research indicates that once the operating frequency deviates from the resonance region, the material reverts to, or even slightly falls below, its “static” performance level, and the dynamic advantages of ACP are lost.

Considering the comprehensive performance across selected parameters, the optimal poling frequency range was [0.57, 1] Hz. The simultaneous peaks of $d_{33}$ and $\varepsilon_{33}^{T}/\varepsilon_{0}$ around 0.6667 Hz, and the peak of $k_{t}$ near 0.8 Hz suggest the presence of two distinct resonance modes. The resonant behavior clearly indicates that the synchronous sharp peaks of $d_{33}$ and $\varepsilon_{33}^{T}/\varepsilon_{0}$ are characteristic of the mechanical resonance of the thickness vibration mode. At the resonance point, mechanical strain and dielectric polarization response are greatly amplified. At higher frequencies, domain switching may not keep pace with the alternating field, leading to partial depolarization and reduced dielectric and piezoelectric responses. As indicated in the data, the performance drops markedly above 1 Hz, suggesting that the effective upper frequency limit for the MACP process is around 1 Hz.

From the entire dataset, $d_{33}$ and $\varepsilon_{33}^{T}/\varepsilon_{0}$ demonstrated a strong positive correlation, which results from a common physical mechanism—mechanical resonance. Resonance amplifies the strain (as a surge in $d_{33}$) while also significantly enhancing domain wall motion and polarization capability under an external field (manifested as a surge in $\varepsilon_{33}^{T}/\varepsilon_{0}$) as different electrical output manifestations of the same resonance phenomenon. $d_{33}$ and $k_{t}$ exhibited a moderate positive correlation, while the correlation between $k_{t}$ and $\varepsilon_{33}^{T}/\varepsilon_{0}$ was weaker, indicating a decoupling. This suggests that maximum output ($d_{33}$, $\varepsilon_{33}^{T}/\varepsilon_{0}$) and the highest efficiency ($k_{t}$) correspond to slightly different optimal domain configurations within the material. At f = 0.6667 Hz, the amplification of output by the resonance effect reaches its maximum, whereas at f = 0.8 Hz, the vibration mode of the domains is likely the more conducive to lossless energy conversion, thereby achieving the higher electromechanical coupling efficiency.

### 3.3. Polarization Electric-Field Amplitude

**Figure 4:**
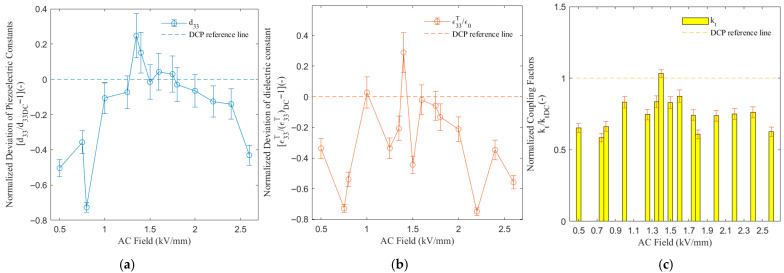
Effect of AC-poling electric field on piezoelectric and dielectric performance in PMN-PT single crystals (frequency: 1 Hz, cycles: 20). (**a**) Evolution of the piezoelectric coefficient $d_{33}$ with AC Field (E_AC_); (**b**) evolution of the dielectric constant $\varepsilon_{33}^{T}/\varepsilon_{0}$ with AC Field (E_AC_); (**c**) evolution of the coupling coefficient $k_{t}$ with AC Field (E_AC_).

Figure 4 shows the ND$d_{33}$, ND$\varepsilon_{33}^{T}/\varepsilon_{0}$, and N$k_{t}$ of the PMN-PT signal crystal with different ACP polarization electric-field amplitudes of the ACP method at 20 cycles and 1 Hz frequency.

At E_AC_ = 0.8 kV/mm, $d_{33}$ exhibited an anomalous trough, while $k_{t}$ and $\varepsilon_{33}^{T}/\varepsilon_{0}$ failed to simultaneously reduce to their minimum values. This may indicate that the domain switching process encounters a specific “bottleneck” or enters an unstable intermediate state at the particular voltage, suggesting that the poling process does not proceed uniformly and likely features critical stages, potentially related to the energy barrier for domain switching at specific voltages. $d_{33}$ reached its peak value at E_AC_ = 1.35 kV/mm, which was 24.79% (0.2479) higher than that of the DCP baseline, representing a significant improvement. Both $\varepsilon_{33}^{T}/\varepsilon_{0}$ and $k_{t}$ achieved their peak values at E_AC_ = 1.4 kV/mm, exceeding the DCP baseline by 28.91% (0.2891) and 3.297% (1.0330), respectively. While $\varepsilon_{33}^{T}/\varepsilon_{0}$ showed a substantial enhancement, $k_{t}$ exhibited only a marginal improvement but maintained a relatively high level comparable to DCP. The E_AC_ in [1.25, 1.4] kV/mm constituted the optimal poling voltage window for achieving well-balanced comprehensive performance.

The peaks of $k_{t}$ and $\varepsilon_{33}^{T}/\varepsilon_{0}$ were highly synchronized, appearing only slightly later than the peak of $d_{33}$. When the E_AC_ was higher than 1.6 kV/mm, these performance parameters began to display a clear oscillatory downward trend. At E_AC_ = 2.2 kV/mm, $\varepsilon_{33}^{T}/\varepsilon_{0}$ showed an anomalously low value, possibly originating from charge injection effects. Eventually, at E_AC_ = 2.6 kV/mm, all parameters degraded to relatively low levels, indicating significant performance deterioration. Excessively high AC voltage damages material performance, which is consistent with the “over-poling” conclusion drawn from the cycle-dependent experiments, confirming that ACP has a definite upper limit.

In this dataset, $d_{33}$ and $\varepsilon_{33}^{T}/\varepsilon_{0}$ demonstrated the strong positive correlation, albeit with local instabilities. The desynchronization at E_AC_ = 0.8 kV/mm, where $d_{33}$ dropped sharply while $\varepsilon_{33}^{T}/\varepsilon_{0}$, is an anomalous point requiring further investigation, potentially stemming from differences in different domain switching modes under the electric field. Their variation trends were highly synchronized, peaking simultaneously in the E_AC_ range of 1.35–1.4 kV/mm and decaying synchronously in the over-poling region (E_AC_ > 1.6 kV/mm). This result indicates that both the quantity and quality of domain alignment simultaneously influence the piezoelectric and dielectric properties.

The synchronization between $k_{t}$ and $d_{33}$, as well as between $k_{t}$ and $\varepsilon_{33}^{T}/\varepsilon_{0}$, were stronger than that observed in the datasets where the number of cycles and frequency were the variables, with their optimal windows almost entirely overlapping. This result indicates that under these (voltage sweep) conditions, optimizing the electromechanical coupling efficiency and optimizing the domain structure for piezoelectric/dielectric constants are part of the same process. The high degree of synchronization, which differs from the “phase shift” observed in the cycle-dependent experiments, demonstrates that different poling control parameters (number of cycles vs. voltage) have distinct influences on the evolution path of the domain structure.

### 3.4. MACP

**Figure 5:**
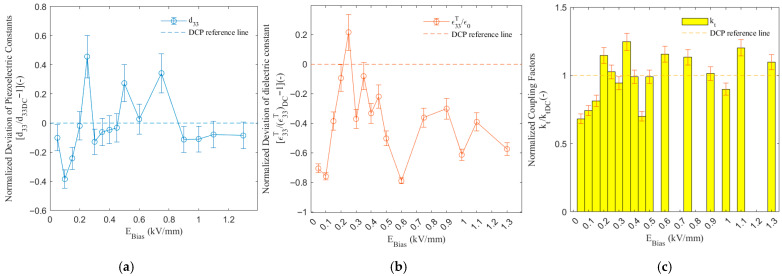
Piezoelectric and dielectric response of MACP-processed PMN-PT single crystals as a function of superimposed DC bias field (E_bias) (1 Hz, 20 cycles, Vpp = 1 kV/mm). (**a**) Evolution of the piezoelectric coefficient $d_{33}$ with bias field ($E_{bia}$); (**b**) evolution of the dielectric constant $\varepsilon_{33}^{T}/\varepsilon_{0}$ with bias field ($E_{bia}$); (**c**) evolution of the coupling coefficient $k_{t}$ with bias field ($E_{bia}$).

Figure 5 shows the ND$d_{33}$, ND$\varepsilon_{33}^{T}/\varepsilon_{0}$, and N$k_{t}$ of the PMN-PT signal crystal with DC-biased potential electric-field amplitude, which is called revised ACP, at 20 cycles, frequency of 1 Hz, and Vpp amplitude of 1 kV/mm.

The $d_{33}$ reached its maximum value at 0.25 kV/mm, with distinct peaks occurring at 0.25, 0.5, and 0.75 kV/mm, exceeding the DCP baseline by 45.67% (0.4567), 27.40% (0.2740), and 34.28% (0.3428), respectively. This exhibited a multi-peak characteristic distinct from the trends observed in Figure 1, Figure 2 and Figure 3. It is inferred that the multi-peak phenomenon reflects the presence of multiple domain switching mechanisms within the material, which are sequentially activated at different bias levels. The $\varepsilon_{33}^{T}/\varepsilon_{0}$ achieved its maximum value at 0.25 kV/mm, surpassing the DCP result by 21.62% (0.2162). The $k_{t}$ significantly exceeded the DCP result at 0.2, 0.35, 0.6, and 1.3 kV/mm, reaching its peak value at $E_{bia}$ = 0.35 kV/mm, which was 24.54% (1.2454) higher than that of the DC baseline.

Compared to the previous datasets, the most distinctive feature of this dataset was the presence of multiple performance peaks, demonstrating pronounced non-monotonicity and complexity. Two clear performance-superior regions were identified: Region I ($E_{bia}$ = 0.2–0.25 kV/mm), where $d_{33}$ and $\varepsilon_{33}^{T}/\varepsilon_{0}$ performed excellently, and Region II ($E_{bia}$ in 0.35–0.6 kV/mm), where the comprehensive performance of $k_{t}$ and $d_{33}$ was favorable. Notably, in the $E_{bia}$ < 0.9 kV/mm range, abrupt troughs were observed in the profiles as a pronounced $d_{33}$ trough at $E_{bia}$ = 0.1 kV/mm, an anomalously low $k_{t}$ value at $E_{bia}$ = 0.45 kV/mm, and an abnormal decrease in $\varepsilon_{33}^{T}/\varepsilon_{0}$ at $E_{bia}$ = 0.6 kV/mm. When $E_{bia}$ > 0.9 kV/mm, all parameters stabilized at a relatively moderate level.

Regarding the inter-variable relationships, $d_{33}$ and $\varepsilon_{33}^{T}/\varepsilon_{0}$ showed a positive correlation as they peaked simultaneously at $E_{bia}$ = 0.25 kV/mm. However, this correlation weakened in other regions, such as at $E_{bia}$ = 0.75 kV/mm, where $d_{33}$ was high but $\varepsilon_{33}^{T}/\varepsilon_{0}$ was moderate, indicating that the bias influences the piezoelectric and dielectric properties through different mechanisms. The $k_{t}$ peaked independently at $E_{bia}$ = 0.35 kV/mm, showing a complete lack of synchronization with both $d_{33}$ and $\varepsilon_{33}^{T}/\varepsilon_{0}$. The independence of $k_{t}$ suggests that the electromechanical coupling coefficient is particularly sensitive to the nanoscale arrangement pattern of the domains and can be decoupled from the macroscopic polarization level (reflected in $d_{33}$ and $\varepsilon_{33}^{T}/\varepsilon_{0}$).

The enhancement in piezoelectric coefficient ($d_{33}$), dielectric permittivity, and electromechanical coupling observed under optimized MACP conditions can be understood through the lens of ferroelectric domain engineering. While direct in situ domain imaging under each poling parameter was not performed here, the macroscopic trends align with and are strongly supported by prior high-resolution microscopy studies on similar PMN-PT crystals. For instance, Xu et al. [13] demonstrated that conventional ACP transforms the domain structure in PMN-0.25PT from irregular, micron-sized domains (characteristic of DCP) into a periodic array of nanoscale stripe domains with significantly increased domain-wall density, as visualized via PFM. This refinement process—directly linked to enhanced $d_{33}$ and $\varepsilon_{33}^{T}/\varepsilon_{0}$—is critically dependent on AC field parameters such as amplitude and frequency.

More recently, Wan et al. provided a detailed investigation of the “overpoling effect” in rhombohedral PMN-0.26PT under ACP [20]. Their PFM images clearly show that optimal ACP leads to fine domains with high wall density, whereas excessive poling cycles cause domain coarsening and reduced wall density, resulting in property degradation. This study establishes a direct visual and quantitative link between domain size, wall density, and macroscopic electromechanical properties under varying poling cycles.

In the context of our MACP approach, the introduction of a DC bias (E_bias ≈ 0.25 kV/mm) superimposed on an optimized AC field (E_AC ≈ 1.35 kV/mm) is interpreted as creating a biased yet dynamic poling environment. This hybrid field is likely to promote a uniform, fine-grained domain pattern with high mobility of non-180° walls, while stabilizing the domain configuration against the coarsening observed in overpoled conventional ACP. Therefore, the optimal parameters identified in this work (0.6667 Hz, 30 cycles) are reasoned to produce a domain architecture that maximizes extrinsic contributions (high $d_{33}$ and $\varepsilon_{33}^{T}/\varepsilon_{0}$) through high domain-wall density, while maintaining strong long-range coupling (good $k_{t}$). This provides a coherent microstructural explanation for the superior integrated performance reported in this study.

The MACP method significantly enhances selected key piezoelectric parameters, which enables precise performance control and directional optimization through multi-dimensional parameters and facilitates the construction of superior microstructures (e.g., engineered domain structures). The DC bias provides stable guidance for the polarization direction, while the AC component promotes refined domain switching and structural optimization. Their synergistic interaction achieves precise control over the domain structure.

### 3.5. Polarization Behavior and Validation Under Optimized MACP Conditions

**Figure 6:**
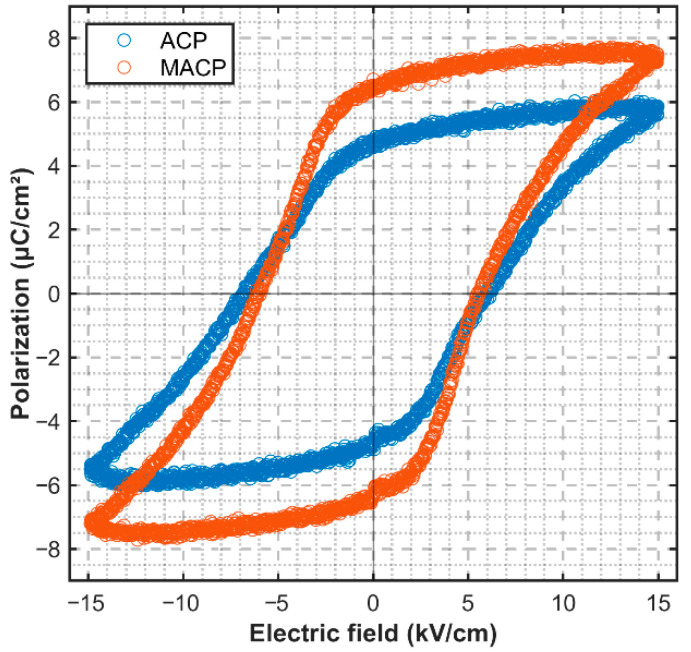
The P-E loops for ACP and MACP of the PMN-PT signal crystals.

The P-E ferroelectric hysteresis loops for ACP and MACP samples are shown in Figure 6 with the thickness direction under the electric field of 15 kV/cm and at the testing frequency of 1 Hz at room temperature. Based on the systematic investigation of the individual effects of E_bia_, E_AC_, frequency, and cycle number on the electromechanical properties (presented in Section 3.1, Section 3.2, Section 3.3 and Section 3.4), an optimized set of Modified AC-Poling (MACP) parameters was identified for enhancing the overall performance. The optimal parameters were determined to be: a DC bias field (E_bia_) of ≈0.25 kV/mm, an AC field amplitude (E_AC_) of ≈1.4 kV/mm, a frequency of 0.6 Hz, and 30 polarization cycles. To validate the synergistic effect of this parameter combination, a dedicated poling experiment was conducted under these exact conditions.

According to the comparative analysis of P-E loops, the sample processed by the MACP method exhibits enhanced ferroelectric properties compared to the one prepared by the standard ACP method. The most direct evidence is the significant increase in saturation polarization (P_s_), which rises from 5.86 μC/cm^2^ for the ACP samples to 7.41 μC/cm^2^ for the MACP samples. This substantial improvement indicates a higher degree of dipole alignment achievable under the modified poling conditions. Correspondingly, the remanent polarization also increased. The positive remanent polarization (P_r_+) improved from 4.60 μC/cm^2^ to 6.67 μC/cm^2^, while the negative remanent polarization (P_r_−) increased in magnitude from −4.88 μC/cm^2^ to −6.25 μC/cm^2^. The slight asymmetry between P_r_+ and P_r_− magnitudes, commonly observed in ferroelectric materials, is attributed to an internal bias field. The obtained P_s_ values are significantly lower than the intrinsic saturation polarization of PMN-PT single crystals (~40–50 µC/cm^2^), indicating that the measurement was conducted under a sub-saturation electric field.

Concurrently, a reduction in the coercive field (E_c_) was achieved. The average coercive field decreased from approximately 5.96 kV/cm for the ACP sample to about 5.53 kV/cm for the MACP sample (specifically, E_c_+ reduced from 5.94 to 5.52 kV/cm, and E_c_− from 5.98 to 5.54 kV/cm). The simultaneous increase in polarization and decrease in coercive field signifies that the MACP process not only enables a higher ultimate polarization state but also facilitates easier domain switching by reducing the energy barrier for dipole reorientation.

A typical room-temperature P-E hysteresis loop measured for the [001]-oriented PMN-0.3PT single crystal exhibits a saturated ferroelectric loop with high squareness, featuring a remanent polarization $P_{r}$ of approximately 32 μC/cm^2^, and a coercive field $E_{c}$ of about 20 kV/mm. These values align with the typical characteristics of relaxor ferroelectric single crystals near the morphotropic phase boundary [22,23]. Notably, the observed high remanent polarization originates directly from the material’s inherent multi-domain structure. Relaxor-based piezoelectric single crystals intended for transducer applications are materials with a multi-domain configuration. According to the IEEE Std 1859-2017 standard [18], this structure enables significant macroscopic polarization switching through domain wall motion under an external electric field, thereby contributing to superior piezoelectric response.

The ferroelectric properties are closely related to the stability of the material’s phase structure. The rhombohedral-to-tetragonal phase transition temperature $T_{RT}$ for PMN-0.3PT lies between 80 and 90 °C. The Curie temperature T_C_ is approximately 140–150 °C [18]. All electrical measurements in this study were conducted at room temperature (~25 °C), well below its $T_{RT}$. Therefore, the material resides in the stable ferroelectric rhombohedral phase, ensuring that the P-E loop measurements reflect intrinsic ferroelectric domain switching behavior, rather than effects from thermal fluctuations near a phase transition.

Regarding the nature of the phase transition, PMN-PT undergoes a typical first-order phase transition around $T_{RT}$, accompanied by abrupt changes in lattice constants and spontaneous polarization [24]. This characteristic is reflected in its P-E loop, manifested as rapid polarization reversal near the coercive field and a relatively steep loop slope. The spontaneous polarization $P_{s}$ is about 2.2 kV/mm for PMN-0.3PT [25]. The high ratio of $P_{s}$ to $P_{r}$ further confirms the material’s favorable ferroelectric switching characteristics. In summary, the P-E loop measurements not only verify the strong ferroelectricity of PMN-0.3PT but also, by combining the parameters ($P_{r}$, $E_{c}$) with its multi-domain structure and first-order transition features, provide a crucial microphysical foundation for understanding its macroscopic piezoelectric and dielectric performance.

The resulting functional properties are summarized in Table 1 and compared with those obtained from conventional DCP and standard ACP (E_bia_ = 0) at the same maximum field (≈1.4 kV/mm). The macroscopic ferroelectric and strain responses are presented in Figure 6, respectively.

As evidenced by the data, the MACP process under the optimized parameters yields a comprehensive enhancement. The piezoelectric coefficient $d_{33}$ reaches 1850 pC/N, which represents an improvement of approximately 95.2% over the standard ACP samples. The obviously increased remanent polarization $P_{r}$ and reduced coercive field $E_{c}$ (Figure 6), consistently point to a more mobile and finely engineered domain structure with a high density of active non-180° domain walls. This optimized state facilitates greater extrinsic contributions under an applied electric field, leading to the superior strain response of 0.49% (Figure 6). Despite the sub-saturation measurement condition, the MACP sample consistently exhibited a higher polarization response within the same electric field window, demonstrating the effectiveness of the modified poling process in enhancing the low-field ferroelectric activity.

## 4. Conclusions

These experiments systematically investigated the effects of four key parameters in ACP—frequency, number of cycles, voltage (E_AC_), and bias ($E_{bia}$)—on the performance of piezoelectric materials ($d_{33}$, $k_{t}$, $\varepsilon_{33}^{T}/\varepsilon_{0}$), and compared the results with traditional DCP. Among them, the cycle-dependent experiments revealed the dynamic evolution process of the domain structure over time and its optimal “time window.” The frequency-dependent experiments uncovered the resonance characteristics of the material’s dynamic response. The AC electric-field strength revealed the energy threshold required to drive domain switching and the hazards of over-poling. Meanwhile, the modified ACP method attempted to directionally optimize material performance on the basis of traditional ACP, providing a new dimension for improving the performance enhancement method for piezoelectric materials. A comprehensive analysis leads to the following core conclusions:

1. Universal “optimal window” effect and non-monotonicity

These PMN-PT performance parameters exhibited strong non-monotonic dependence on the four poling parameters, meaning there exists a clear “optimal poling window,” contrary to the notion that “higher/longer parameters are better.” The frequency window is around 0.57 Hz to 1.0 Hz. The cycle window is around 23 to 30 cycles. The voltage window (E_AC_) is around 1.25 kV/mm to 1.4 kV/mm. The bias window ($E_{bia}$) exhibits dual-region characteristics (Region I at around 0.2–0.25 kV/mm; Region II at around 0.35–0.6 kV/mm).

Hazards of over-poling need to be attended to. Once the optimal windows are exceeded (e.g., cycles > 30, E_AC_ > 1.6 kV/mm), these performance parameters degrade significantly, providing a clear warning for process control.

2. Inter-variable correlations reveal the physical mechanisms of domain evolution

$d_{33}$ and $\varepsilon_{33}^{T}/\varepsilon_{0}$ exhibit a strong positive correlation in most cases, as they both depend on the quantity of domains aligned along the poling direction. Their simultaneous peaks in both the frequency- and cycle-dependent experiments indicate a common origin from the mechanical resonance of the thickness vibration mode.

In contrast, the relationship between $k_{t}$ and $d_{33}$/$\varepsilon_{33}^{T}/\varepsilon_{0}$ is more complex, showing synergistic, competitive, and differentiated behaviors. In the cycle-dependent experiments, the peak of $k_{t}$ occurs earlier than those of $d_{33}$ and $\varepsilon_{33}^{T}/\varepsilon_{0}$. This phase shift suggests a staged poling process: the domain structure quality is first optimized (enhancing efficiency $k_{t}$), followed by an increase in domain quantity (boosting output $d_{33}$ and $\varepsilon_{33}^{T}/\varepsilon_{0}$).

3. Synergistic Mechanism of the Optimized AC/DC Poling Conditions

The optimal poling condition, characterized by a relatively small DC bias (E_bia_ ≈ 0.25 kV/mm) superimposed on a larger AC field (E_AC_ ≈ 1.35 kV/mm), can be understood as a synergistic two-step domain engineering process. This specific field ratio is crucial for maximizing performance. The sub-coercive DC component (E_bia_ < E_c_) establishes a preferential orientation [26,27] within the poly-domain state, effectively breaking the symmetry and creating a biased energy landscape without fully saturating the polarization [28,29]. This pre-conditioning lowers the activation energy for the subsequent switching of favorable non-180° domain variants.

The significantly larger AC field then plays the primary role in dynamic domain refinement. It actively drives repeated domain wall motion and switching cycles within this biased framework. The AC component does not work against a fully saturated, single-domain state but rather selectively activates and pumps domain walls in the pre-oriented, multi-domain matrix [30]. This process promotes the proliferation of mobile non-180° domain walls (primarily 90° walls in tetragonal phases) and refines the domain structure into a dense, periodic pattern.

The synergy arises because the small DC bias guides the AC-driven process toward a more desirable and stable domain configuration, while the large AC field efficiently populates this configuration with a high density of active walls. This cooperative effect simultaneously enhances the extrinsic contributions (high $d_{33}$ and $\varepsilon_{33}^{T}/\varepsilon_{0}$ from wall motion) and optimizes the macroscopic coupling efficiency (improved $k_{t}$), explaining why this particular AC/DC field ratio yields the best overall electromechanical performance.

4. ACP with bias—The key to performance customization

The multi-peak phenomenon observed in the bias data is the most complex and informative manifestation in ACP. The MACP method fails to merely combine the effects of ACP and DCP through simple superposition. Instead, this integration produces an unexpected multi-peak response, while also enabling $k_{t}$ to maintain an elevated level surpassing DCP as the bias increases. It indicates that the synergistic effects of the DC bias field and the AC field can selectively activate different domain switching modes. These results contribute to achieving high $d_{33}$ around an $E_{bia}$ of 0.25 kV/mm, suitable for sensors and actuators, and achieve high $k_{t}$ around an $E_{bia}$ of 0.35 kV/mm, suitable for resonators and energy harvesters. The research results provide a powerful processing method for the fabrication of “functionally customized” piezoelectric devices.

Building upon the systematically mapped optimal parameter windows, this work proposes a performance-customized processing route via MACP, where the domain structure can be strategically engineered by targeting distinct parameter sets. For high-output and sensing applications requiring maximized piezoelectric charge output, the recommended protocol centers on a DC bias of ~0.25 kV/mm combined with an AC field at ~0.67 Hz, ~1.35 kV/mm, and applied for 30 cycles; this configuration prioritizes the quantity of aligned domains and strong lattice strain, driving peak $d_{33}$ enhancements over 45%. Conversely, for high-efficiency resonant devices where superior electromechanical coupling is critical, the optimal strategy employs a DC bias of ~0.35 kV/mm with an AC field at ~0.8 Hz, ~1.4 kV/mm, and 23 cycles, thereby refining the domain configuration into a nano-scale pattern that favors efficient energy conversion, achieving a $k_{t}$ value ~24.5% above the DC-poled benchmark. This tailored framework enables the fabrication of devices with application-specific performance maxima directly derived from the identified experimental optima.

This research not only confirms the significant advantages of ACP in enhancing the performance of piezoelectric materials. More importantly, it maps out a complete optimization framework for the poling process. By precisely controlling the four dimensions—frequency, number of cycles, voltage, and bias—it is possible to achieve “precision sculpting” of the material’s micro-domain structure, thereby breaking through the performance bottlenecks of traditional DCP.

Future work should focus on multi-parameter co-optimization—i.e., jointly optimizing bias, voltage, and number of cycles at the determined optimal frequency—to achieve unprecedented comprehensive performance and to provide clear and reliable poling process specifications for different application scenarios. Furthermore, employing imaging techniques to analyze the microscopic principles (e.g., PFM or under linearly polarized light) of the observed phenomena from a domain perspective is recommended.

## Figures and Tables

**Table 1:** Comparison of functional properties for PMN-PT crystals under different poling conditions.

**Poling Method**	$d_{33}(pC/N)$	$P_{r}$ $+(\mu C/cm^{2})$	$P_{s}(\mu C/cm^{2})$	$E_{c}(kV/mm)$
ACP	948	4.60	5.86	5.96
MACP	1850	6.67	7.41	5.53

## Data Availability

The datasets generated and analyzed during the current study are available from the corresponding author upon reasonable request.

## Abbreviations

The following abbreviations are used in this manuscript:

ACP	Alternating-current poling
DCP	Direct-current poling
MACP	Modified alternating-current poling
PMN-PT	Pb(Mg_1/3_Nb_2/3_)O_3_-PbTiO_3_
PFM	Piezoresponse force microscopy
